# Neural Basis of Impaired Cognitive Flexibility in Patients with Anorexia Nervosa

**DOI:** 10.1371/journal.pone.0061108

**Published:** 2013-05-10

**Authors:** Yasuhiro Sato, Naohiro Saito, Atsushi Utsumi, Emiko Aizawa, Tomotaka Shoji, Masahiro Izumiyama, Hajime Mushiake, Michio Hongo, Shin Fukudo

**Affiliations:** 1 Department of Psychosomatic Medicine, Tohoku University Hospital, Sendai, Miyagi, Japan; 2 Department of Clinical Neuroscience, Yamagata University Graduate School of Medical Science, Yamagata City, Yamagata, Japan; 3 Stress Care Clinic Lumeto, Natori, Miyagi, Japan; 4 Department of Mental Disorder Research, National Institute of Neuroscience, National Center of Neurology and Psychiatry, Tokyo, Japan; 5 Sendai Nakae Hospital, Sendai, Miyagi, Japan; 6 Department of Physiology, Tohoku University Graduate School of Medicine, Sendai, Miyagi, Japan; 7 Kurokawa Hospital, Taiwa-cho, Miyagi, Japan; 8 Department of Comprehensive Medicine, Tohoku University Hospital, Sendai, Miyagi, Japan; 9 Department of Behavioral Medicine, Tohoku University Graduate School of Medicine, Sendai, Miyagi, Japan; Chiba University Center for Forensic Mental Health, Japan

## Abstract

**Background:**

Impaired cognitive flexibility in anorexia nervosa (AN) causes clinical problems and makes the disease hard to treat, but its neural basis has yet to be fully elucidated. The purpose of this study was to evaluate the brain activity of individuals with AN while performing a task requiring cognitive flexibility on the Wisconsin Card Sorting Test (WCST), which is one of the most frequently used neurocognitive measures of cognitive flexibility and problem-solving ability.

**Methods:**

Participants were 15 female AN patients and 15 age- and intelligence quotient-matched healthy control women. Participants completed the WCST while their brain activity was measured by functional magnetic resonance imaging during the task. Brain activation in response to set shifting error feedback and the correlation between such brain activity and set shifting performance were analyzed.

**Results:**

The correct rate on the WCST was significantly poorer for AN patients than for controls. Patients showed poorer activity in the right ventrolateral prefrontal cortex and bilateral parahippocampal cortex on set shifting than controls. Controls showed a positive correlation between correct rate and ventrolateral prefrontal activity in response to set shifting whereas patients did not.

**Conclusion:**

These findings suggest dysfunction of the ventrolateral prefrontal cortex and parahippocampal cortex as a cause of impaired cognitive flexibility in AN patients.

## Introduction

Patients with anorexia nervosa (AN) have a cognitive deficit relating to own body weight and shape [1]. Their perception of body shape is seriously distorted and they refuse to maintain a minimally normal weight because of an intense fear of gaining weight. AN patients strictly limit their food intake (restrictive type: ANR) and/or binge eat and purge (binge-purge type: ANBP), and in many cases, their thoughts are occupied with food. Accordingly, a number of neuroimaging studies on AN have focused on the role of body image [2]–[6] or food stimuli [7]–[9], but they have produced inconsistent results and other pathogenic factors need to be examined in greater detail in AN research.

Cognitive impairment in AN extends beyond symptoms concerning body image and food to involve visuospatial ability [10]–[13], attention [12]–[14], memory [11], [13]–[16], and cognitive flexibility [17]–[23]. Cognitive flexibility is the ability to alter a behavior in response to changes in the situation, and impaired cognitive flexibility is considered to be a risk factor of AN [20], [24]. The deficit causes behavioral rigidity which leads to maintenance of symptoms [20], [25] and resistance to treatment. Impaired cognitive flexibility in AN patients has been found to have no correlation with body weight [17], [23], [26]. Recovered AN patients have also shown cognitive flexibility impairment [23], [27], and interestingly unaffected sisters of AN patients have shown poorer cognitive flexibility than healthy controls [23], [26]. These findings suggest that impaired cognitive flexibility in AN patients is not a temporary state due to starvation but is a trait characteristic. One study has reported that AN patients without comorbid depression showed intact cognitive flexibility during several cognitive tasks [28]. As many as 86% of AN patients are reported to have lifetime comorbid depressive disorder [29], 64% to have anxiety disorders [30], and 21.7% to have at least one personality disorder [31]. However, the nature of the relationship between cognitive flexibility and comorbidities in AN patients remains to be elucidated.

In recent years, cognitive functions not associated with food or body image have started to be evaluated in individuals with AN, using functional magnetic resonance imaging (fMRI) [32]–[36]. Recovered AN patients were found to have higher caudate activity during a monetary reward task than healthy controls, and while healthy controls responded differently to reward and penalty feedback in the anterior ventral striatum, recovered AN patients had almost the same response to both conditions [32]. Adolescents with AN showed significantly higher activation than healthy controls in the temporal and parietal areas during a working memory task, a difference that disappeared after weight recovery [35]. Moreover, patients with ANBP showed greater activation than controls in the bilateral precentral gyri, anterior cingulate cortex (ACC), and superior and middle temporal gyri in a response inhibition task, while patients with ANR showed poorer activity than those with ANBP in the hypothalamus and right dorsolateral prefrontal cortex (DLPFC) [36]. Recovered AN patients showed poorer medial prefrontal activity than controls during a more difficult response inhibition task [34]. Zastrow et al. reported that AN patients had a significantly higher error rate in behavioral response shifting during a target detection task focused on cognitive and behavioral flexibility [33]. During the behavioral response shifting, the patients showed less activation than controls in the left and right thalamus, ventral striatum, ACC, and sensorimotor brain regions but higher activation in the frontal and parietal regions. Hypoactivity of anterior cingulate-striato-thalamic loop appeared to be associated with impaired behavioral response shifting, but no deficit in cognitive set shifting was seen during the task. Any definitive evidence of the pathogenesis of AN remains elusive, however.

The Wisconsin Card Sorting Test (WCST) [37] is one of the most widely used neurocognitive measures to evaluate cognitive flexibility. A lesion in the prefrontal cortex can cause difficulties on the WCST [38]–[40], and neuroimaging studies have demonstrated activation of the frontostriatal circuit during the WCST [41]–[45]. The lateral prefrontal cortex (LPFC) is also a key area that is activated during cognitive flexibility tasks [46]. Healthy participants have shown significant LPFC activation in the condition requiring set shifting during the WCST [42], [44], [45]. AN patients have been reported by several authors to show poor performance on the WCST [17], [20]–[22], [47], but there have been no brain imaging studies conducted on individuals with AN while completing the WCST.

We hypothesized that AN patients would show poor performance and hypoactivity in the LPFC during the WCST. To test this hypothesis and try to elucidate the neural basis of impaired cognitive flexibility in AN patients, we administered the WCST to AN patients and controls while measuring blood oxygen level dependent (BOLD) signals of the brain with fMRI.

## Participants and Methods

### 1. Participants

Forty-eight right-handed women participated in this study. Twenty-one individuals who fulfilled the DSM-IV-TR criteria for AN [1] —11 with ANR and 10 with ANBP—were recruited from outpatients and inpatients at Tohoku University Hospital. Handedness was determined by the Edinburgh Handedness Inventory [48]. We excluded individuals with claustrophobia, visual impairment including defect in color perception, metallic implant, lifetime presence of head trauma and/or neurological disease, or life-threatening physical condition. Those with past/present Axis I or II psychiatric disorders were also excluded, but AN patients with depressive disorder, anxiety disorder, or personality disorder, which are highly prevalent comorbidities in AN patients, were included in this study to reflect the typical clinical situation. None of the healthy controls took medication. Given that cognitive flexibility is impaired by major tranquilizers [49], that some cognitive functions are impaired by both acute [50] and long-term [51] benzodiazepine administration, but that serotonin selective reuptake inhibitor (SSRI) minimally affects cognitive task performance [52], we also excluded the patients who took major tranquilizer/benzodiazepine anxiolytics but included those who took SSRIs. On this basis, the following AN patients were excluded: one who had mental retardation, and 2 with ANR and 3 with ANBP who took a major tranquilizer and/or benzodiazepine anxiolytic and were later excluded. Participant screening and diagnosis were performed by board certified specialists of the Japanese Society of Psychosomatic Medicine at Tohoku University Hospital based on medical interview according to the DSM-IV-TR. This left 15 AN patients as participants in this study—9 ANR patients and 6 ANBP patients. Four of the 15 patients were taking a selective serotonin reuptake inhibitor (SSRI) and took it even on the day of fMRI session (3 diagnosed with depression and the remaining patient diagnosed with obsessive-compulsive disorder). One patient was diagnosed as having borderline personality disorder. Twenty-seven healthy controls were registered for this study and we selected 15 who were intelligent quotient (IQ)- and age-matched to the patients. Although all 15 had served female controls in our previous fMRI study on the brain activity of patients with irritable bowel syndrome [53], the hypothesis and target disease of the two studies are different, and our control data used in the previous study was composed of 15 men combined with 15 women, and therefore the women's control data have not been reported previously. All controls were within the normal weight range (body mass index (BMI) 18–23 kg/m^2^) and all had been recruited by advertisement from among university students, were free from medication, and reported no history (lifetime diagnosis) of psychiatric disease.

### 2. Ethics statement

This study was approved by the Ethics Committee of the Tohoku University School of Medicine and all participants provided written informed consent to participate. All participants were judged to have the ability to give consent through a medical interview by board certified specialists of the Japanese Society of Psychosomatic Medicine at Tohoku University Hospital. Next of kin, caretakers or guardians consented on the behalf of those participants under the age of 20.

### 3. Psychological assessment

The 26-item Eating Attitudes Test (EAT-26) [54] was administered to all participants to evaluate their eating behavior and severity of eating disorder. The Wechsler Adult Intelligence Scale-Revised was administered to evaluate intelligence and exclude the possible influence of hidden impairment of intellectual functioning. Full-scale intelligence quotient (IQ), Verbal IQ, and performance IQ scores were calculated. The Minnesota Multiphasic Personality Inventory (MMPI) Japanese version [55] was administered to all participants. Depressive and anxiety disorders are prevalent comorbidities of AN patients, as described above. On the MMPI, an elevated score on scale 2, depression (D), indicates feelings of depression and sadness, and an elevated score on scale 7, psychasthenia (Pt), indicates generalized feelings of anxiety and discomfort [56]. Therefore, we report the T scores for MMPI scale 2 and 7.

### 4. Task

**Figure 1:**
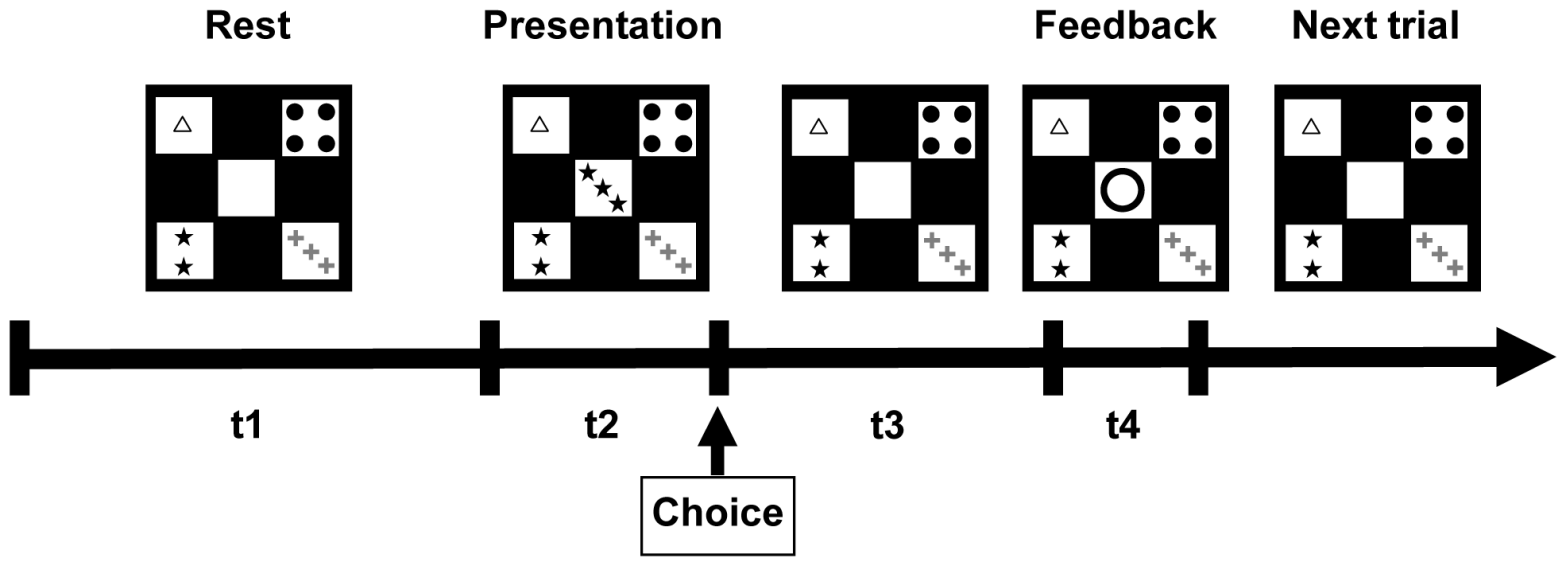
A trial sequence of the Wisconsin Card Sorting Test. t1: 3 or 4 sec, t2 (reaction time): ≤2 sec, t3: (3 or 4) - t2 sec, t4: 1 sec.

The WCST was administered in the same manner as in our previous fMRI study on patients with irritable bowel syndrome [53]. In brief, the WCST task presentation was computerized with the pictures rear-projected onto the screen of the MRI scanner. Four fixed reference cards were displayed at the corners of the screen. Participants looked at the screen through a periscope mirror mounted on the head coil. The graphic forms on the four cards all had the elements of color, shape, and number: “one red star”, “two green squares”, “three yellow crosses”, and “four blue circles”. A test card was presented in the center of the screen. The participant held a switch box in the right hand and clicked a button to select one of the four reference cards that she judged to have the same kind of element of the test card. Three different matching rules were used in the judgment: color matching, shape matching, and number matching. Participants were not informed about the matching rules applied during the test. An “O” symbol was used as feedback for a correct response (**Fig. 1**) and an “X” symbol for an error. When five consecutive responses were correctly judged, the rule was changed without notice. The participant would realize a rule change when receiving the error symbol and would need to change the response strategy; that is, shift the cognitive set, a function strongly correlated with WCST performance [57]. Problems in set shifting may manifest as impaired cognitive flexibility [58]. The participants were fully trained in the task on a personal computer prior to scanning. One trial consisted of a period of rest, task presentation, card selection, and feedback. A total of 128 trials were conducted in a single session lasting 17 minutes and 6 seconds. We programmed a rule change after five consecutive correct responses were made and a jitter during the resting period and when waiting for feedback (**Fig. 1**). To determine task performance, we calculated from the responses the correct rate of selection, total error rate, perseverative error rate, and non-perseverative error rate. Perseverative error, an index of persistency, was defined as a response following a rule that had been correct earlier but was incorrect later [38].

### 5. Image acquisition

We acquired images on a 1.5 T Siemens Magnetom Symphony® MRI scanner (Siemens, Erlangen, Germany) using a standard two-channel head coil at Sendai Nakae Hospital. A time-course series of 342 volumes was acquired with T2*-weighted gradient-echo-planer imaging sequences depicting BOLD contrasts during the task. Each volume consisted of 30 slices parallel to the anterior commissure-posterior commissure line in ascending order. Repetition time was 100 ms per slice (total repetition time 3000 ms) with an echo time of 62 ms and a flip angle of 90°. The field of view was 192 mm and the matrix size was 64×64, giving a voxel dimension of 3.0×3.0×5.0 mm with no gaps. Structural scans were acquired using a T1-weighted gradient echo pulse sequence, which facilitated localization.

### 6. fMRI data analysis

Image processing and statistical analysis was performed using Statistical Parametric Mapping (SPM 5; (Wellcome Department of Cognitive Neurology, London, UK, http://www.fil.ion.ucl.ac.uk/spm/) implemented in Matlab, version 7 (Mathworks Inc., Natick, MA). The first two volumes of the fMRI scans were discarded because of unsteady magnetization. Each set of functional volumes was realigned to the first scan with allowed motion limited to ±1 mm translation and ±1 degree rotation. The images were corrected for differences in slice acquisition timing and were spatially normalized to a standard template based on the Montreal Neurological Institute reference brain. Volumes were smoothed using an 8-mm full width, half maximum Gaussian filter. Low-frequency signal drifts were removed using a 128-s high-pass filter. As prefrontal cortex activity is reported to be increased in response to negative or positive feedback and to be greater than the response to matching [45], [59], we analyzed the brain activity in response to feedback. In the first level analysis, a General Linear Model (GLM) was fitted to data for a single participant using six regressors of interest, namely set shifting error feedback and first to fifth feedback in a series of five consecutive correct responses (referred to hereafter as “first correct feedback”), which were modeled as a stick function and convolved with the canonical hemodynamic response function. Responses to set shifting error feedback and first correct feedback were considered for analysis. Both set shifting error feedback and first correct feedback signaled to the participant that the situation had changed. The first correct feedback signaled to the participant to keep the strategy (cognitive set keeping), whereas the set shifting error feedback signaled to shift the cognitive set. Thus, with this contrast we could extract brain activity specific to set shifting. For the group analysis, contrast images from each participant were entered into a hierarchical model equivalent to a random-effects model. Group activation maps used a height threshold of p<0.001 (uncorrected) and clusters were considered statistically significant at cluster-level p<0.05, corrected for multiple comparisons across the whole brain. We used a one-way ANOVA to evaluate brain activity in response to set shifting error feedback vs. first correct feedback among the ANR, ANBP and control participants. Voxel-wise significance was set at p<0.05 (family wise error (FWE) corrected). Post-hoc t-tests between all combinations of two out of the three groups were done using Bonferroni correction. Voxel-wise significance was set at p<0.00033 and cluster-wise significance at p<0.017. Anatomical labeling of peak coordinates was done using Talairach Client ver. 2.42 (http://www.talairach.org/). Correlational analysis was performed for task performance, demographic data, and brain activity in the regions that showed a significant difference between the AN patients and control groups. We calculated the mean contrast value of a spherical region of interest centered at the peak voxel of the cluster and with a diameter of 6 mm. MarsBar [60], a toolbox for SPM, was used for this purpose. Values of p<0.05 were considered significant.

### 7. Statistical analysis

We used JMP Pro® ver.9 (SAS Institute Inc., Cary, NC) for statistical analysis of demographic and WCST task performance data. The AN and HC groups were compared with Student's two sample t-test. A one-side t-test was performed for task performance as previous studies on the WCST reported that AN patients showed significantly poor performance [17], [20]–[22]. We performed correlation analysis between demographic data and WCST performance for each group (HC and all AN patients). To compare demographic/clinical characteristics and WCST performance of the two subgroups of AN (ANR and ANBP) and HC, we used a Kruskal-Wallis one-way analysis of variance (ANOVA). Significance was set at p<0.05. Multiple comparisons between all combinations of two out of the three groups were done using the Steel-Dwass method, with significance set at p<0.05.

## Results

### 1. Demographic data and behavior results

There were no differences in age or IQ between the groups. BMI was significantly lower in patients than in controls (p<0.0001, **Table 1**). Duration of AN was 3.6±3.7 (mean ± SD) years, with no significant difference between the two AN subgroups (ANR 3.6±3.6 years, ANBP 3.5±4.1 years) (Table 1). EAT-26 score was significantly higher in AN patients than in controls (p = 0.0002, **Table 1**). AN patients had significantly higher T scores on MMPI scale 2 (depression, p = 0.0465) and 7 (anxiety, p = 0.0276) than the controls (**Table 1**). AN patients showed a significantly lower correct rate on the WCST than controls (p = 0.0420, **Table 2**). None of the other performance data differed between the controls and AN patients. Neither BMI nor MMPI scale 2 and 7 scores were correlated with WCST performance in the controls or AN patients. One-way ANOVA of the demographic/clinical characteristics and WCST performance for the ANR, ANBP, and control participants showed no significant results. Multiple comparison revealed the ANR and ANBP patients had significantly lower BMI and EAT-26 score than the controls (**Table 1**). Multiple comparison showed no other significant results.

**Table 1:** Demographic and Clinical Characteristics of Patients with Anorexia Nervosa and Healthy Controls.

Characteristic	AN (n = 15)	ANR (n = 9)	ANBP (n = 6)	HC (n = 15)	p
Age (years)	23±7	21±5	26±9	22±3	0.4997
Body mass index (kg/m^2^)	14.6±1.5	14.7±1.9^a^	14.5±0.7^a^	20.6±1.2	<0.0001**
duration (years)	3.6±3.7	3.6±3.6	3.5±4.1	-	-
Full-scale IQ	97.8±13.7	99.6±15.4	95.2±11.4	104.9±11.3	0.1344
Verbal IQ	97.5±12.7	100.4±13.7	93.2±10.6	104.5±12.8	0.1440
Performance IQ	98.0±13.9	98.6±15.9	97.2±11.8	104.5±12.6	0.1942
MMPI Scale 2	65.8±17.3	68.9±17.6	61.2±17.3	55.2±8.8	0.0465*
MMPI Scale 7	65.2±14.2	66.1±15.3	63.8±13.7	55.6±6.5	0.0276*
EAT-26	25.3±15.9	21.3±12.1^a^	31.2+20.2^a^	4.3±4.6	0.0002**

AN: all anorexia nervosa patients, ANR: restrictive anorexia nervosa patients, ANBP: binge-purge anorexia nervosa patients, HC: healthy controls, IQ: intelligent quatient, MMPI: Minnesota Multiphasic Personality Inventory, EAT-26: 26-item Eating Attitudes Test. Mean ± SD,

* p<0.05,

** p<0.01, two sample t-test between all AN patients and HC.

a : p<0.01, multiple comparison vs. HC using the Steel-Dwass method.

**Table 2:** Task Performance of the Wisconsin Card Sorting Test.

Performance (%)	AN (n = 15)	ANR (n = 9)	ANBP (n = 6)	HC (n = 15)	p
Correct rate	70.0±8.8	72.1±9.0	66.8±8.2	74.7±4.8	0.0420*
Total error rate	23.5±4.0	21.7±2.6	26.2±4.4	23.6±4.7	0.4743
Perseverative error rate	6.8±5.6	6.1±7.1	7.9±1.9	6.4±4.3	0.3995
Non-perseverative error rate	16.7±4.3	15.6±4.8	18.2±3.0	17.2±1.1	0.3105

AN: all anorexia nervosa patients, ANR: restrictive anorexia nervosa patients, ANBP: binge-purge anorexia nervosa patients,HC: healthy controls. Mean ± SD,

* p<0.05, two sample t-test between all AN patients and HC.

### 2. Imaging results

**Figure 2:**
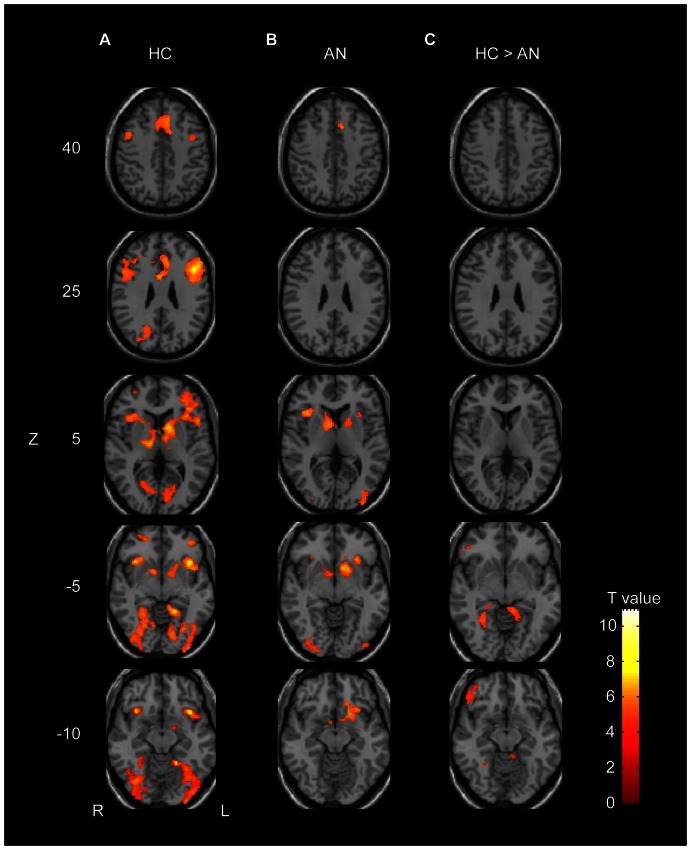
Brain activity on set shifting error feedback vs. activity on first correct feedback. L: Left, R: Right. z: z coordinate of Talairach space. AN: all anorexia nervosa patients, HC: healthy controls. One sample t-test for a single group test, two sample t-test for group comparison between HC and all AN patients. Significance levels were voxel-wise p<0.001 (uncorrected) and cluster-wise p<0.05 (corrected).

Clusters of significant brain activation are shown in **Figure 2** **and** **Table 3** **–** **8**. In the tables, cerebellar activations are not shown because activity in the cerebral cortical and subcortical regions is the focus of this study. Healthy controls showed significantly more activity in the DLPFC and ventrolateral prefrontal cortex (VLPFC), cingulate cortex, insula, occipital cortex, parahippocampal cortex (PHC), and basal ganglia of both hemispheres at set shifting than at first correct feedback (**Table 3**, **Fig. 2A**). The all-AN patient group showed significantly more activity in response to set shifting in bilateral occipital cortices, bilateral insula, bilateral basal ganglia, and bilateral cerebellum than to first correct feedback (**Table 4**, **Fig. 2B**). ANR patients showed significantly more activity in response to set shifting in the putamen, insula, and caudate head (Table 5). ANBP patients showed no significant activation. In the group comparison between the all-AN patients and controls, AN patients showed poorer activity than the controls in the right VLPFC (BA47) and bilateral PHC (**Table 6**, **Fig. 2C**). AN patients did not show higher brain activity than the controls in any brain region. Whole brain one-way ANOVA among the two subgroups of AN patients and controls showed significant clusters in the cingulate cortex, putamen, and insula (Table 7). ANBP patients showed significantly lower activity in right VLPFC than controls (Table 8). Multiple comparison showed no other significant results.

**Table 3:** Brain Activity of Healthy Controls (n = 15) on Set Shifting Error Feedback vs. Activity on First Correct Feedback of Five Consecutive Corrects.

L/R	Area	BA	x	y	z	T value	voxel	p
L	Insula	13	−35	14	−5	10.91	4379	0.000
L	Parahippocampal gyrus	19	−17	−48	−4	10.29	3793	0.000
L	Cingulate gyrus	32, 24	−9	22	33	10.18	2034	0.000
R	Insula	13	34	18	0	7.27	1944	0.000
R	Anterior cingulate	10	21	45	−3	5.56	228	0.002

L/R: Left/Right, BA: Brodmann area, x, y, z: Talairach coordinates of the peak voxel. 1 voxel = 2 mm×2 mm×2 mm. p: cluster-wise p value (corrected). Significance levels were voxel-wise p<0.001 (uncorrected) and cluster-wise p<0.05 (corrected), one sample t-test.

**Table 4:** Brain Activity of Anorexia Nervosa Patients (n = 15) on Set Shifting Error Feedback vs. Activity on First Correct Feedback of Five Consecutive Corrects.

L/R	Area	BA	x	y	z	T value	voxel	p
L	Putamen	-	−17	3	4	8.86	838	0.000
R	Insula	13	34	17	7	7.94	265	0.000
R	Globus pallidus	-	11	0	3	6.55	435	0.000
L	Middle occipital gyrus	19, 18	−34	−92	8	6.28	229	0.000
L	Middle frontal gyrus	8	−7	18	44	6.10	95	0.020
R	Middle occipital gyrus	18, 19	29	−89	3	5.22	205	0.000

L/R: Left/Right, BA: Brodmann area, x, y, z: Talairach coordinates of the peak voxel. 1 voxel = 2 mm×2 mm×2 mm. p: cluster-wise corrected p value (corrected). Significance levels were voxel-wise p<0.001 (uncorrected) and cluster-wise p<0.05 (corrected), one sample t-test.

**Table 5:** Brain Activity of Restrictive Anorexia Nervosa Patients (n = 9) on Set Shifting Error Feedback vs. Activity on First Correct Feedback of Five Consecutive Corrects.

L/R	Area	BA	x	y	z	T score	Voxel	p
L	Claustrum	-	−30	21	−1	9.05	101	0.000
L	Putamen	-	−16	8	0	8.02	118	0.000
R	Insula	13	36	20	3	6.81	104	0.000
R	Caudate head	-	8	4	3	6.58	96	0.001

L/R: Left/Right, BA: Brodmann area, x, y, z: Talairach coordinates of the peak voxel. 1 voxel = 2 mm×2 mm×2 mm. p: cluster-wise corrected p value. Significance levels were voxel-wise p<0.001 (uncorrected) and cluster-wise p<0.05 (corrected), one sample t-test.

**Table 6:** Group Comparison between Anorexia Nervosa Patients (n = 15) and Healthy Controls (n = 15) for Brain Activity on Set Shifting Error Feedback vs. Activity on First Correct Feedback of Five Consecutive Corrects.

L/R	Area	BA	x	y	z	T value	voxel	p
R	Parahippocampal gyrus	19, 27	27	−50	0	5.44	161	0.022
L	Parahippocampal gyrus	30	−9	−40	0	5.16	152	0.028
R	Inrerior Frontal gyrus	47	48	38	−13	5.15	199	0.008

L/R: Left/Right, BA: Brodmann area, x, y, z: Talairach coordinates of the peak voxel. 1 voxel = 2 mm×2 mm×2 mm. p: cluster-wise p value (corrected). Significance levels were voxel-wise p<0.001 (uncorrected) and cluster-wise p<0.05 (corrected), two sample t-test.

**Table 7:** One-way ANOVA of Brain Activity among Healthy Control Women (n = 15), Restrictive Anorexia Nervosa Patients (n = 9), and Binge-Purge Anorexia Nervosa Patients (n = 6) on Set Shifting Error Feedback vs. Activity on First Correct Feedback of Five Consecutive Corrects.

L/R	Area	BA	x	y	z	F score	voxel	p
L	Cingulate gyrus	32	−8	23	34	30.6	30	0.002
L	Putamen	-	−16	8	0	29.32	66	0.002
R	Insula	13	38	19	−1	25.08	17	0.012

L/R: Left/Right, BA: Brodmann area, x, y, z: Talairach coordinates of the peak voxel. 1 voxel = 2 mm×2 mm×2 mm. p: voxel-wise p value (Family Wise Error corrected). Significance level was p<0.05, one-way ANOVA. Degree of freedom = [3.0, 27, 0].

**Table 8:** Post-hoc Group Comparison between Binge-purge Anorexia Nervosa Patients (n = 6) and Healthy Controls (n = 15) for Brain Activity on Set Shifting Error Feedback vs. Activity on First Correct Feedback of Five Consecutive Corrects.

L/R	Area	BA	x	y	z	T score	voxel	p
R	Inferior frontal gyrus	47	48	38	−15	5.48	116	0.006

L/R: Left/Right, BA: Brodmann area, x, y, z: Talairach coordinates of the peak voxel. 1 voxel = 2 mm×2 mm×2 mm. p: cluster-wise p value (corrected). Significance levels were voxel-wise p<0.00033 (Bonferroni corrected) and cluster-wise p<0.017 (Bonferroni corrected), post-hoc two sample t-test.

**Figure 3:**
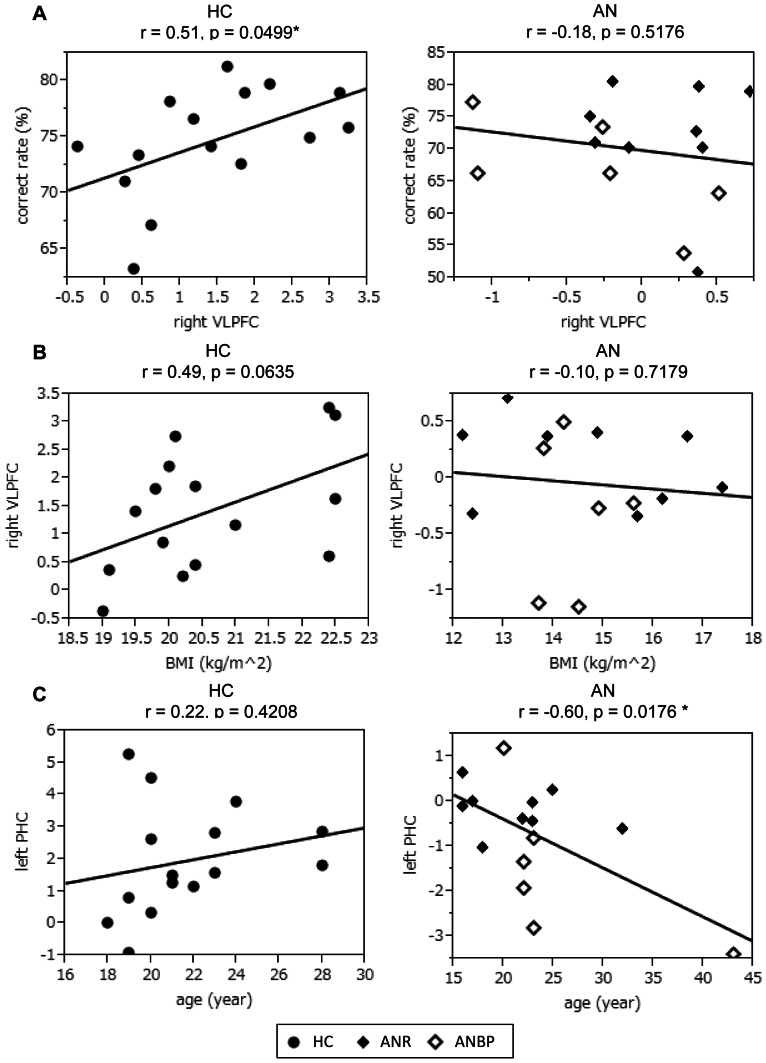
Scatter plots for correlation between brain activity and the Wisconsin Card Sorting Test (WCST) performance or demographic data. A. Correlation between right ventrolateral prefrontal cortex (VLPFC) activity and correct rate of WCST. B. Correlation between body mass index (BMI) and right VLPFC activity. C. Correlation between age and left parahippocampal cortex (PHC) activity. Brain activity: mean contrast value on set shifting feedback vs. 1^st^ correct feedback. AN: anorexia nervosa patients, HC: healthy controls. r: coefficient of correlation, * p<0.05.

Individual mean contrast values for right VLPFC activation on set shifting error feedback greater than activity on first correct feedback showed a positive correlation with correct rate in controls (r = 0.51, p = 0.0499) but not in AN patients (r = −0.18, p = 0.5176) (**Fig. 3A**). In controls, the higher the right VLPFC activity was in response to set shifting feedback, the higher the correct rate achieved. In contrast, in AN patients, right VLPFC activity was not related to the correct rate. Neither controls nor AN patients showed a significant correlation between BMI and activity in any brain region of interest (**Fig. 3B** shows a correlation between BMI and right VLPFC activity). A significant negative correlation was found between age and left PHC activity in AN patients (r = −0.60, p = 0.0176) but not in controls (r = 0.23, p = 0.4208) (**Fig. 3C**). Left PHC activity in AN lessened with advancing age.

## Discussion

This study showed that the correct rate on the WCST was poorer for AN patients than healthy controls, but there was no difference in perseverative error rate between the two groups. This result is consistent with an earlier study that found adolescents with ANR showed more total errors on the WCST than IQ-matched controls, but the same perseverative errors as the controls [61]. Other earlier WCST studies comparing AN patients with controls at the same educational level showed that AN patients had poorer performance in regard to total error and perseverative error [17], [20]–[22], [47], but the IQ of participants in these studies were not matched. One large study reported that AN patients were more perseverant than healthy controls on the WCST [62], but neither educational level nor IQ was mentioned in the study. Given our findings, if the IQ of healthy controls had been matched with that of AN patients in these previous studies, the patients would likely have had fewer perseverative errors on the WCST. BMI showed no correlation with WCST performance in either the controls or AN patients in the present study, which is consistent with the findings of earlier studies on cognitive flexibility in AN patients [17], [23], [26]. These results suggest that impaired cognitive flexibility in AN patients is not a state due to starvation but is a trait of the disease.

Our results for the controls showed set shifting-related activity in the LPFC, cingulate cortex, PHC, and basal ganglia in both hemispheres. These findings are consistent with earlier neuroimaging studies on WCST performance in normal healthy participants [63]. By contrast, our control participants showed no activity in the parietal region, an area shown to be activated in many WCST neuroimaging studies [45], [59], [64]–[66]. This conflicting finding was probably because we subtracted the response to first correct feedback from the response to set shifting error feedback. Parietal cortex activation has been reported for both positive and negative feedback [45], and the magnitude of response to set shifting error feedback in the parietal cortex may be similar to that for first correct feedback.

AN patients showed higher activation in the cingulate cortex and striatum, insula, and occipital cortex during set shifting in the present study. Several earlier studies of AN patients reported higher activity in these regions in a variety of conditions [5], [32], [35], [67], [68]. Adolescent AN patients before treatment showed higher activity in the occipital and cingulate cortices during a working memory task than they did after treatment [35]. Recovered AN women showed greater hemodynamic activation in the caudate than control women [32], and AN patients showed greater activation in the insula during a body image task [5], [67], [68]. However, in the lateral frontal regions, which are thought to be crucial for set shifting [46], our AN patients showed no activation.

The AN patients in our study showed a poorer response in the right VLPFC (BA47) and bilateral PHC (BA19, 30). ANBP patients also showed significantly lower activity than controls in the right VLPFC, a region which was also highlighted in the group comparison results between controls and all AN patients. This result is different from that of an earlier study on set shifting in AN patients [33] which followed a paradigm by Shafritz et al. focusing on the difference between behavioral response shifting and cognitive set shifting [46]. Shafritz et al. observed activation in the DLPFC, ACC, and inferior parietal lobe during behavioral response shifting, but in the VLPFC, ACC, and striatum during cognitive set shifting. In a study by Zastrow et al., AN patients showed a poorer response than controls in the ACC, ventral striatum, and thalamus on behavioral response shifting, but the patients demonstrated no deficit in cognitive set shifting [33]. Our AN patients showed hypoactivity in the VLPFC in response to set shifting feedback, but full activation of the ACC and striatum, other areas involved in cognitive set shifting. The difference between the two studies probably derives from the difference in paradigms. The WCST requires participants to create a new cognitive set on the rule change, whereas the task used in the earlier study was designed to present a new behavioral strategy overtly, which required a passive behavioral change (i.e. behavioral response shifting). The covert rule change on the WCST in our study meant that participants made cognitive set shifts voluntarily, which was why we decided to use the WCST.

The VLPFC is thought to be one of the regions critical for set shifting ability. This region, where a group difference was observed in our study, did not show significant activation in healthy participants on the single-group statistical test, but correlational analysis showed that controls with better performance on the WCST showed better brain activity in this region. Some previous studies have reported activation in the VLPFC on the WCST [45], [59]. VLPFC activity was shown to be increased specifically in response to negative feedback, prompting a change in the task [45]. Shifts in cognitive set were found to be mediated by the VLPFC, ACC, and striatum [46], and to occur specifically in these areas with the inhibition of previously acquired stimulus–response rules and the acquisition of new stimulus–response associations [59]. Activity change in the right VLPFC was demonstrated on stopping the response to the previously relevant stimulus and shifting it in response to the newly relevant stimulus [69]. In our study, the VLPFC in AN patients was less activated in response to set shifting error feedback, which may explain the impaired cognitive flexibility reported in AN patients.

Bilateral activation of the PHC was observed in our healthy controls. In the group comparison, the controls showed greater activation of the PHC than AN patients. Poor parahippocampal activity seen in our AN patients is consistent with some earlier studies. Lower parahippocampal activity was observed in AN patients viewing their own body [4], and healthy women showed more activity in the left PHC in response to negative words about body image, whereas AN patients show no activity in the PHC [6]. In an electroencephalography study on healthy participants, delta wave activity was increased in the PHC, PFC, ACC, and other cortical-subcortical regions during the WCST [70]. Regional cerebral blood flow of healthy participants, evaluated with positron emission tomography, was also shown to be increased in the left PHC during the modified card sorting test [71]. The PHC is thought to be part of a widespread neural network involved in efficient WCST performance [63] and appears to be activated during future event simulation [72]. Many patients with eating disorder have difficulty in talking about recovery (i.e., a better future) or to have less ability to imagine for themselves [73]. Our result may explain AN patients' deficit in future imagination, a function that is important for problem solving.

We found no correlation between BMI and brain activity in any of three regions (right VLPFC, right PHC, and left PHC) in which AN patients showed hypoactivity in our study.Furthermore, all three such regions showed no correlation with other characteristics of AN symptomatology, namely duration and EAT-26 score. Our results support the speculation that impaired cognitive flexibility in AN patients is a trait, not a state due to starvation. Left parahippocampal activation on set shifting showed negative correlation with age in AN patients. This may reflect chronicity of the disease. Further investigation is needed to clarify the effect of chronicity.

Our study has several limitations. First, the small sample size limits statistical power. It did not allow us to investigate the difference between the two subtypes of AN (ANR and ANBP) Whole brain one-way ANOVA among the ANR, ANBP and control participants showed significant clusters in the cingulate cortex, putamen, and insula. However, post-hoc analysis showed no difference in these regions, perhaps because of the small sample size. Investigation with a larger sample size is needed. Second, we could not completely exclude the influence of psychiatric comorbidity (e.g., depression, anxiety, obsessive-compulsive disorder, or borderline personality disorder) on our results. In regard to the possible effect of depression, our AN patients showed significantly higher MMPI depression score than controls. One study has reported that set shifting ability during several cognitive tasks including the WCST was intact in AN patients withoutcomorbid depression [28]. In our study, none of the WCST performance scores were correlated with the depression scale score of AN patients or controls. Moreover, AN patients' hypoactive regions in response to set shifting feedback showed no correlation with depression score. We also found AN patients had a higher MMPI psychasthenia score than controls. There was no correlation between MMPI psychasthenia scale score and WCST performance, and psychasthenia score had no correlation with brain activity where AN patients showed hypoactivity in response to set shifting feedback.. Thus, depression or anxiety at the least unlikely affected our results. Third, a possible confounding factor in our study is that 4 patients took an SSRI. However, blockade of the serotonin transporter by SSRIs has been documented for the midbrain, striatum, amygdala and other subcortical areas [74] but not for the LPFC or PHC. Further studies are needed to clarify the effects of regional brain dysfunction in AN patients.

In conclusion, women AN patients showed poorer WCST performance than healthy control women. They also showed set shifting-specific hypoactivity in the VLPFC and PHC. Such hypoactivity in the brain of AN patients may be responsible for their impaired cognitive flexibility.
